# Does homologous reinfection drive multiple-wave influenza outbreaks? Accounting for immunodynamics in epidemiological models^☆^

**DOI:** 10.1016/j.epidem.2013.09.003

**Published:** 2013-12

**Authors:** A. Camacho, B. Cazelles

**Affiliations:** a Eco-Evolution Mathématique, UMR 7625, CNRS-UPMC-ENS, 75230 Paris Cedex 05, France; b Department of Infectious Disease Epidemiology, London School of Hygiene and Tropical Medicine, London, United Kingdom; c UMMISCO UMI 209 IRD-UPMC, F-93142 Bondy, France

**Keywords:** Influenza, Mechanistic modelling, Multiple-wave outbreak, Pandemic, Primary immune response, Reinfection

## Abstract

Epidemiological models of influenza transmission usually assume that recovered individuals instantly develop a fully protective immunity against the infecting strain. However, recent studies have highlighted host heterogeneity in the development of this immune response, characterized by delay and even absence of protection, that could lead to homologous reinfection (HR). Here, we investigate how these immunological mechanisms at the individual level shape the epidemiological dynamics at the population level. In particular, because HR was observed during the successive waves of past pandemics, we assess its role in driving multiple-wave influenza outbreaks. We develop a novel mechanistic model accounting for host heterogeneity in the immune response. Immunological parameters are inferred by fitting our dynamical model to a two-wave influenza epidemic that occurred on the remote island of Tristan da Cunha (TdC) in 1971, and during which HR occurred in 92 of 284 islanders. We then explore the dynamics predicted by our model for various population settings. We find that our model can explain HR over both short (e.g. week) and long (e.g. month) time-scales, as reported during past pandemics. In particular, our results reveal that the HR wave on TdC was a natural consequence of the exceptional contact configuration and high susceptibility of this small and isolated community. By contrast, in larger, less mixed and partially protected populations, HR alone cannot generate multiple-wave outbreaks. However, in the latter case, we find that a significant proportion of infected hosts would remain unprotected at the end of the pandemic season and should therefore benefit from vaccination. Crucially, we show that failing to account for these unprotected individuals can lead to large underestimation of the magnitude of the first post-pandemic season. These results are relevant in the context of the 2009 A/H1N1 influenza post-pandemic era.

## Highlights

• We model the primary immune responses to influenza infection in humans.
• We examine the interplay between immunological and epidemiological dynamics.
• The model explains cases of homologous reinfection reported during past pandemics.
• Three epidemic profiles can arise depending on the degree of population mixing.
• A substantial proportion of infected host would remain unprotected after the 2009 influenza pandemic.

## Introduction

Mathematical models of infectious diseases often rely on a compartmental description in order to reduce the population diversity to a few key characteristics which are relevant to the infection under consideration. An extensively used model for influenza infection is of susceptible-exposed-infectious-removed (SEIR) form: after exposure to the virus, susceptible hosts (S) pass through an exposed state (E) of latent infection, become infectious (I) and are finally removed (R) from the infectious pool as they simultaneously recover (or die) and acquire permanent protection against the infecting strain. The SEIR model was particularly successful during the 2009 pandemic in estimating the key transmission parameters of the novel H1N1 virus (nH1N1) (Fraser et al., 2009) and assessing the effectiveness of alternative vaccination strategies (Baguelin et al., 2010).

Nevertheless, proper consideration of the primary immune response, which occurs on the first exposure to a novel influenza virus, motivates a more accurate description of the different stages from recovery to development of long-term protective immunity. Indeed, the primary immune response to influenza in humans operates on two different time scales. Usually, the viral load is cleared by the innate and cellular immune responses within a few days following infection (Woodland, 2003), thus leading to recovery of infected hosts. By contrast, the humoral (antibody-mediated) immune response, which provides long-term protection against the infecting strain as well as closely related strains (Fairlie-Clarke et al., 2008), takes several weeks to become efficient (Cox et al., 2004; Miller et al., 2010; Baguelin et al., 2011). Finally, at the population level, there is host heterogeneity in the development of this long-term protective immunity as some individuals show high antibody titres shortly after recovery whereas some other fail to reach a protective level (Cox et al., 2004; Miller et al., 2010; Chen et al., 2010; Hung et al., 2010; Chan et al., 2011).

In a recent study, Camacho et al. (2011) showed that a precise account of these host heterogenities was necessary to explain the reinfection episodes reported during the “natural experiment” of Tristan da Cunha (TdC), a remote island that underwent a two-wave A/H3N2 influenza epidemic in 1971 (Mantle and Tyrrell, 1973). More precisely, in the next few days that followed its introduction, the virus spread rapidly throughout the whole island population and after three weeks of propagation, 273 (96%) of 284 islanders had been infected. However, while the epidemic was nearing its end, several recovered islanders developed a second illness, thus initiating the second epidemic wave during which at least 92 (32%) islanders were reinfected (see section “Data” for more details). The main finding of Camacho et al. (2011) is that, among six biologically realistic reinfection mechanisms, only two could be retained: some hosts with either a delayed or deficient humoral immune response to the primary influenza infection were reinfected following rapid re-exposure to the same strain. This historical event illustrates that host heterogeneity at the individual level can not only lead to HR but also shape the epidemiological dynamics by triggering a second epidemic wave.

Historically, multiple-wave outbreaks and rapid reinfections have commonly been observed during influenza pandemics. The most striking example remains the “Spanish” influenza pandemic of 1918–1919 that occurred in three waves (Taubenberger and Morens, 2006) and during which several reinfection episodes were reported, sometimes in proportions similar to that of the 1971 TdC epidemic (Medical Department of the Local Government Board, 1919; Ministry Of Health, 1920; Barry et al., 2008). However, the three epidemic waves in 1918–1919 were spread out over 9 months (Taubenberger and Morens, 2006) whereas the two-wave epidemic on TdC lasted only 59 days (Mantle and Tyrrell, 1973). Accordingly, the time-scale between successive infections in the same individual was of the order of months during the pandemic whereas it was of the order of a few weeks for the TdC islanders, thus questioning their common underlying biological mechanisms. More recently, many populations experienced a spring and a fall waves during the 2009 pandemic and several cases of HR were virologically confirmed (Perez et al., 2010; Kim et al., 2010). Most of these HR episodes occurred within 2–3 weeks following recovery, a time-scale similar to that observed among the TdC islanders. However, both infection and HR occurred over the same epidemic wave in 2009 whereas they were separated across both waves in TdC, thus questioning the role of HR in driving multiple-wave outbreaks.

Overall, these observations call for clarification of the significance of HR and its role in driving multiple-wave outbreaks during pandemics. In particular, to what extent a better consideration of the immunological dynamics may be important in epidemiological models of influenza pandemics? In order to investigate these issues, we propose to explore and characterize the interplay between the immunological and epidemiological dynamics of a novel influenza virus. We start by defining an extended SEIR model accounting for the primary immune response to influenza and its inherent host heterogeneity. Using a maximum-likelihood (ML) approach, we confront our mechanistic model with the time-series of the daily incidence counts of the 1971 TdC epidemic and obtain ML estimates for the key immunological parameters. This analysis also reveals the exceptional setting of the TdC population and lead us to explore the impact of HR on the epidemiological dynamics for various population settings. We conclude with a discussion on the role of HR in the current post-pandemic era.

## Materials and methods

### The primary immune response to influenza infection in humans

A multi-pronged innate (McGill et al., 2009) and adaptive (Brown et al., 2004) immune response has been described for clearing influenza infection. The innate response is the first to be activated and plays a key role through its ability to control early viral replication and to promote and regulate the virus-specific adaptive immune response (McGill et al., 2009). The adaptive response itself may be broken into two critical sub-components: (i) the cellular immune response by which antigen-specific cytotoxic T lymphocytes (CTLs) eliminate infected cells and thus prevent viral release; and (ii) the humoral immune response by which serum and mucosal antibodies efficiently neutralize the virus (as explained in Text S1 the separation between serum and mucosal antibodies is not necessary for our study). Antibodies can remain detectable for years after infection and prevent reinfection by the same strain as well as by sufficiently cross-reactive variants (Fairlie-Clarke et al., 2008). Genetic variation in any of these immune components might determine whether or how rapidly an individual develops protective immunity following influenza infection.

**Fig. 1:**
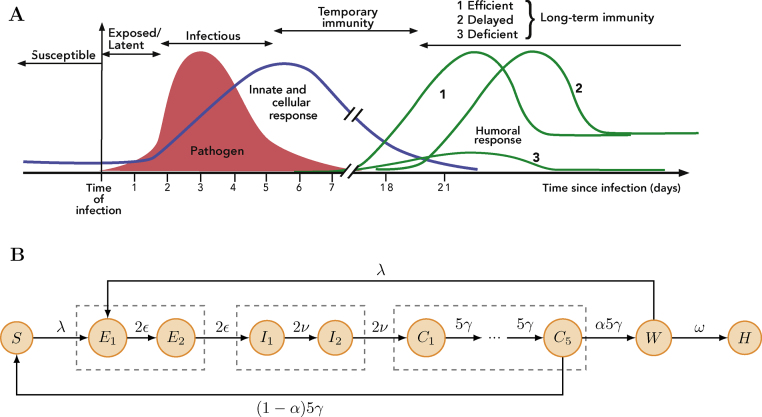
Mechanistic modelling of the primary immune response to influenza. (A) Schematized dynamics of the viral load as well as the innate and adaptive immune responses, as described in section “The primary immune response to influenza infection in humans”. (B) The SEICWH model. The six immunological stages are *S*: susceptible, *E*: exposed, *I*: clinically ill and infectious, *C*: temporarily protected by the cellular response, *W*: temporarily susceptible, *H*: protected on the long-term by the humoral response. The number of sub-compartments in each immunological stages corresponds to the shape of the Erlang distribution for the residence time in this stage (see section “Mechanistic modelling”). The infection force is *λ* = *β*(*I*_1_ + *I*_2_)/*Ω*. A description of the parameters can be found in Table 2. The transition rates used to stochastically simulate the model are provided in Table 1. The set of ordinary differential equations used for deterministic simulations can be found in Text S3.

As schematized in Fig. 1A, it is important to note that, during a primary influenza infection, the innate and cellular responses (blue curve) play the key role in viral clearance whereas neutralizing antibodies (green curve) are generated later and do not play a significant role unless the viral load is high and sustained (Woodland, 2003). The primary CTL response is detectable in blood after 6–14 days whereas the neutralizing antibody response peaks at 4–6 weeks (Cox et al., 2004). Critically, the CTL response is down-regulated after viral clearance (Woodland, 2003), disappears by day 21 post-infection (Cox et al., 2004) and is followed by a state of immunological “memory” with antigen-specific T cells. The memory cells cannot prevent HR as well as specific antibodies could, but they can reduce the severity of the disease (Woodland, 2003). Finally, it has been reported that a serum or mucosal antibody response cannot be detected in approximately 10 to 20% of subjects after natural influenza infection (Cox et al., 2004; Tamura and Kurata, 2004; Miller et al., 2010; Chen et al., 2010; Hung et al., 2010; Chan et al., 2011).

### Mechanistic modelling

Fig. 1B shows the SEICWH model which extends the classical SEIR model to account for the dynamics and host heterogeneity of the primary immune response to influenza in humans. Following recovery, hosts remain temporarily protected against HR thanks to the cellular response. Accordingly, they enter the C stage (cellular protection). Then, following down-regulation of the CTL response, the humoral response has a probability *α* to reach a level sufficient to protect against HR. In this case, recovered hosts enter the H stage (humoral protection) but otherwise they remain unprotected and re-enter the susceptible pool (S). Finally, in order to account for potential delay between completion of CTL contraction and full development of the neutralizing antibody response, recovered hosts pass through a time window of susceptibility (W) before entering the *H* stage. Crucially, while in the W stage, individuals can be reinfected following re-exposure to the same strain

In order to account for host heterogeneity in the development of the immune response, we use a stochastic framework to simulate the durations of the successive immunological stages. Defining *τ*_*E*_, *τ*_*I*_, *τ*_*C*_ and *τ*_*W*_ as the times spent by an infected host in the indexed immunological stages, we assume that these four random variables follow independent Erlang distributions with shapes *k*_*E*_, *k*_*I*_, *k*_*C*_ and *k*_*W*_ and means *ϵ*^−1^, *ν*^−1^, *γ*^−1^ and *ω*^−1^, respectively. Erlang distribution with mean *m* and shape *k* is modelled by *k* consecutive sub-stages, each being exponentially distributed with mean *m*/*k*. As illustrated in Fig. S1 the flexibility of the Erlang distribution ranges from the exponential (*k* = 1) to Gaussian-like (*k* ≫ 1) distributions. In particular, whenever *k* > 1 the memory-less property of the exponential distribution is lost, thus providing more realistic distribution for biological processes with delays such as recovery or contraction of the CTL response (Wearing et al., 2005). In Fig. 1B, as well as in the rest of the paper, we use *k*_*E*_ = *k*_*I*_ = 2, *k*_*W*_ = 1 and *k*_*C*_ = 5. Justification of these values is provided in section “Parameter inference via maximum likelihood”.

Finally, regarding disease transmission, we make the standard assumptions of a well mixed, isolated and constant size (= *Ω*) population, as well as a constant contact rate (= *β*) among individuals. These simplifications permit us (i) to focus on the direct impacts of the immunological mechanisms on the epidemic dynamics; and (ii) to rapidly assess these impacts for various population settings, i.e. contact rates. We refer to the last section of this paper for a discussion on the inclusion of further refinements in the transmission mechanisms.

### Data

The data counts are clinical records based on symptom observation and were drawn from the notes taken during the regular work of the local practice who visited all but three houses during the course of the epidemic in TdC (Mantle and Tyrrell, 1973). In addition, blood sample of 11 individuals provided serological confirmation of the circulation of A/H3N2 on the island, a subtype to which the TdC population had never been exposed before 1971. We refer to the paper of Mantle and Tyrrell (1973) for a detailed description of the 1971 data set as well as to the paper of Camacho et al. (2011) for a summary of the influenza experiences in TdC before 1971.

**Fig. 2:**
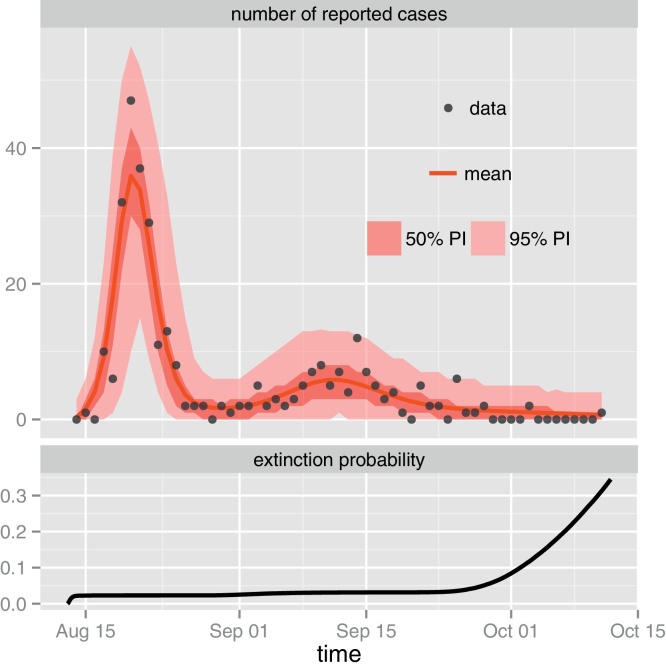
Detailed analysis of the 10^5^ realizations of the stochastic SEICWH model for the 1971 TdC epidemic using Gillespie's algorithm (Gillespie, 1977). Upper panel: original incidence data (black dots) and expected incidence (red line) conditioned on non-extinction under the best fit model together with 50 and 95 percentile intervals (red envelopes) due to demographic stochasticity. This figure demonstrates that the best fit of the SEICWH model captures the shape and the dynamics of the data. Lower panel: time course of the extinction probability *p*(*t*) defined as the probability that the epidemic has faded out by time *t* and estimated by the proportion of fade-out realizations at time *t*. (For interpretation of the references to color in this figure legend, the reader is referred to the web version of the article.)

We note that the data are not available at the individual level. However, since the data set consists of 312 cases for 284 islanders some individuals must appear twice in the data counts. More precisely, in their original article, Mantle and Tyrrell (1973) states that among the 284 islanders 273 were infected at least once whereas 92 were reinfected. Unfortunately, only 312 of the 365 total cases were recorded with daily precision and were included in their data set (Mantle and Tyrrell, 1973) which is reproduced in Fig. 2 (black dots). As such, we can only conclude that at least 49 HR cases appear in the data.

### Simulation

Our aim is (i) to fit the SEICWH model to the 1971 TdC epidemic in order to estimate the immunological parameters (*ϵ*, *ν*, *γ*, *ω*, *α*) and (ii) to explore the model dynamics for various population settings. For this purpose, we used both stochastic and deterministic simulations of the SEICWH model, as we now explain.

In a previous study, Camacho et al. (2011) showed that, given the small population of TdC, demographic stochasticity should be taken into account when fitting a mechanistic model to the 1971 TdC epidemic. This is because the risk of epidemic fade-out during the trough between waves depends on the model parameters and must therefore be accounted for when maximizing the likelihood over the parameter space. Accordingly, we exclusively resorted to stochastic simulations to fit the 1971 TdC epidemic. In the stochastic SEICWH model, the number of individuals in each immunological (sub-)stage is a discrete random variable and a possible state of the population at time *t* is defined by the random vector

$$\text{X}(t)=(S(t),E_{1:2}(t),I_{1:2}(t),C_{1:5}(t),W(t),H(t))$$

where *C*_1:5_ ≡ *C*_1_, …, *C*_5_ (and similarly for *I*_1:2_ and *E*_1:2_). The state of the population at time *t* is therefore a realization

$$\text{x}(t)=(s(t),e_{1:2}(t),i_{1:2}(t),c_{1:5}(t),w(t),h(t))$$

of **X**(*t*). The time course of **x**(*t*) is led by the possible transitions described in Table 1 and was simulated using Gillespie's exact algorithm (Gillespie, 1977).

Our second aim was to explore the dynamics of the SEICWH model over a wide range of parameter values. In this context, stochastic simulations become computationally intensive and one is tempted to resort on deterministic simulations for the sake of efficiency. However, this approximation is acceptable only as one controls that the stochastic effects should remain negligible. Accordingly, we assessed that the inter-wave extinction probability remains negligible (*p* < 10^−3^) in the parameter range explored (see Text S3), thus justifying the use of a deterministic approximation. In the deterministic SEICWH model, the state of the population **x**(*t*) becomes a continuous variable governed by a set of ordinary differential equations (given in Text S3) that can be obtained by the large population limit (*Ω*→ ∞) of the stochastic process (Kurtz, 1971). These equations were numerically integrated using the fourth-order Runge-Kutta routine of the GSL library (Galassi et al., 2010).

### Parameter inference via maximum likelihood

For a time series *y*_1:*T*_ of *T* successive observations and a state-space model with parameter vector *θ*, the likelihood is given by $L(\theta )=P(y_{1:T}|\theta )$. For our stochastic model, the likelihood is analytically intractable and we resorted to an iterated filtering procedure which converges to the ML parameter estimate (*θ*_ML_) to the incidence data (Ionides et al., 2006) (code available upon request). In short, this inference framework only requires: (i) an algorithm to simulate the stochastic model; and (ii) an observation process to link the model-predicted incidence (i.e. the daily number of new hosts entering the infectious class *I*_1_) to the daily incidence counts reported in the data set. Following Camacho et al. (2011), we used Gillespie's algorithm for model simulation and a Poisson process, whose reporting rate *ρ* was also inferred, for observation. We performed log-likelihood profiles in order to check convergence to the maximum likelihood and to calculate 95% confidence intervals (CI_95%_) for parameter estimates.

A detailed description of the inference procedure can be found in the Supplementary Material of Camacho et al. (2011). In particular, it is shown that, in contrast to the other parameters, inference of the shape parameters *k*_*E*_, *k*_*I*_, *k*_*C*_ and *k*_*W*_ is computationally too expensive. To tackle this issue, we followed Wearing et al. (2005) who fitted a SEIR model to an influenza epidemic in a boarding school and obtained the best fit for *k*_*E*_ = *k*_*I*_ = 2. Then, we performed sensitivity analyses on *k*_*C*_ and *k*_*W*_ and found that, whatever the value of *k*_*C*_, the likelihood was maximized when *k*_*W*_ = 1 (results not shown). Finally, we performed a log-likelihood profile on *k*_*C*_ and found the maximum at *k*_*C*_ = 5. In the remainder of this paper we therefore fix *k*_*E*_ = *k*_*I*_ = 2, *k*_*W*_ = 1 and *k*_*C*_ = 5 and obtain the model of Fig. 1B.

### Quantities of epidemiological interest

Our detailed description of the different immunological stages allows us to derive several quantities of epidemiological interest. We denote by *τ* the time elapsed since the start of the infectious period and define the following probabilities:

• $P_{1}(\tau )=P[\tau_{I}>\tau ]=1-\int_{0}^{\tau }f_{I}(t)dt$, the probability that, at time *τ*, the host is still infectious,
• $P_{2}(\tau )=P[\tau_{I}+\tau_{C}>\tau ]=1-\int_{0}^{\tau }f_{\mathrm{IC}}(t)dt$, the probability that, at time *τ*, the host is still temporarily protected against HR thanks to the innate and cellular immunity,
• $P_{3}(\tau )=P[\tau_{I}+\tau_{C}+\tau_{W}<\tau ]=\alpha \int_{0}^{\tau }f_{\mathrm{ICW}}(t)dt$, the probability that, at time *τ*, the host is already protected on the long-term against HR thanks to the humoral immunity,
• *P*_4_(*τ*) = 1 − *P*_2_(*τ*) − *P*_3_(*τ*), the probability that, at time *τ*, the host is unprotected and can potentially suffer from HR if re-exposed,

where *f*_*IC*_ is the probability density function of *τ*_*I*_ + *τ*_*C*_ and similarly for *f*_*ICW*_. The probability distributions *P*_1–4_ were computed via Monte-Carlo integration.

These probability distributions can be compared with empirical ones obtained from volunteer challenge studies (Carrat et al., 2008) or population surveys during natural infections (Miller et al., 2010; Hung et al., 2010; Chan et al., 2011). In a recent study, Baguelin et al. (2011) fitted a Weibull distribution to a data set consisting of 115 time intervals to seroconversion that were obtained from a serological survey during the second wave of the 2009 A/H1N1 pandemic in the UK (Miller et al., 2010). More precisely, the authors defined the seroconversion interval of each individual as the time taken since symptom onset to reach an hemagglutination-inhibition (HI) titre of ≥32 (Baguelin et al., 2011). Historically, HI assay has been considered to be the gold standard for evaluation of the humoral serum response, with an HI titre of ≥32 considered as a surrogate marker for recent infection during the 2009 pandemic (Hardelid et al., 2010). We investigated the link between seroconversion and efficient protection by comparing the Weibull distribution obtained by Baguelin et al. (2011) to the distribution *P*_3_(*τ*) under our ML parameter estimates. For this purpose we implicitly assume that the time of influenza symptom onset coincides with the start of the infectious period.

In order to assess the impact of HR on the epidemiological dynamics, we define the fraction of the population that was (i) infected at least once (*F*_*I*._), (ii) reinfected (*F*_*II*._) and iii) infected only once but remains unprotected (*F*_*IS*_) at the end of the epidemic. We note however that the model presented in Fig. 1B does not allow us to compute these fractions since it only tracks the epidemiological status of individuals (e.g. susceptible, infectious, protected) rather than their infection and immunological histories. For instance, it is impossible to distinguish between those infected only once and those reinfected (they all pass through the same stages), nor between those susceptible who escaped infection and those unprotected who escaped reinfection (they all end in the *S* class). To tackle this issue, we developed a version of the SEICWH model that allows us to track both the infection and the immunological histories of individuals and used this model to compute *F*_*I*._, *F*_*II*._ and *F*_*IS*_. Since this model is of much higher dimension than that of Fig. 1B, we refer to Text S4 for a more detailed description.

Finally, we define the daily inflow of unprotected hosts $U_{d}$ by counting the number of recovered hosts who lose their temporary immunity conferred by the cellular protection (i.e. leave the *C*_5_ compartment) during day *d*, independently of the outcome of their humoral response.

### Exploration

Human communities differ from each other in their contact structure. We seek to characterize the interplay between the immunological and epidemiological dynamics of the SEICWH model for various contact rates (*β*) ranging from highly to less mixed populations. We keep the immunological parameters constant and equal to those inferred from the 1971 TdC epidemic. We can thus express changes in the contact rate in terms of the more meaningful basic reproduction number (*R*_0_ = *β*/*ν*).

We then focus on the first post-pandemic influenza seasons and seek to determine under which conditions a subsequent variant to the pandemic strain can break population herd immunity, thus leading to a typical seasonal epidemic. Rapid evolution of the pandemic strain through mutations mainly results in changes of its antigenic properties and/or its transmissibility. Other properties such as the duration of the infectious period could also evolve but we keep them constant for simplicity. As such, evolution of the transmissibility translates into a difference Δ*R*_0_ between the basic reproduction number of the mutated variant and that of the pandemic strain. On the other hand, antigenic evolution is modelled through an immune escape factor *σ* ∈ [0, 1] which corresponds to the proportion of antigenic properties of the mutated variant that differs from the pandemic strain. For instance, *σ* = 0 means that both the pandemic and the mutant strain share the same antigenic properties. Finally, we assume that immune escape translates into cross-immunity by reducing the susceptibility against infection by the new variant by a multiplicative factor 1 − *σ*. As such, individuals who have developed a protective humoral response to the pandemic strain are partially protected against infection by the mutant strain whereas those who escaped infection or remained unprotected at the end of the pandemic season are fully susceptible. As detailed in Text S5, previous empirical and modelling studies suggest a relative increase of the transmissibility Δ*R*_0_/*R*_0_ ∈ [0, 1] as well as an immune escape *σ* ∈ [0, 0.5] for a post-pandemic variant. In the following, we explore these parameter ranges.

## Results

### Parameter inference

ML estimates and CI_95%_ for the parameter set are presented in Table 2. Our estimates reveal the exceptional epidemiological context of the 1971 epidemic, in the small and fully isolated TdC community, characterized by a high contact rate among the islanders (*R*_0_ = 11.78, CI_95%_ = [7.7–25.5]), as well as a very low level of pre-existing immunity at the beginning of the epidemic (*S*_0_/Ω ≈ 98 %, [97–99]), the origin of which we speculate upon in Text S6. ML estimates of the generation time (average time between primary and secondary cases: 3.34 days [2.53–4.7]) and of the reporting rate in the data counts (due to asymptomatic infections and observation errors: 71%, [62–82]) are in good agreement with those previously published (Carrat et al., 2008). Similarly, we find that 17%, [0–51] of the infected islanders did not mount an efficient humoral immune response, which is in the range of the estimates available in the literature (Cox et al., 2004; Miller et al., 2010; Chen et al., 2010). Finally, ML estimates of the duration of the short-term protection (13.37 days [10.37–16.31]) that follows recovery and of the window of susceptibility (2.75 days [0–6.03]) that precedes the establishment of a long-term humoral protection are both in good agreement with the timings of the completion of the CTLs contraction (Cowling et al., 2012) and the peak of neutralizing antibodies (see section “The primary immune response to influenza infection in humans”).

We note that the CI_95%_ of the mean window of susceptibility (*ω*^−1^) contains 0 which could suggest a more parsimonious model. However, as we show in Text S7, the broad CI_95%_ of *ω*^−1^ is due to a strong correlation with the parameter *α* (the probability to mount an efficient humoral response). In particular, the lower bound *ω*^−1^ = 0 corresponds to values of *α*∼ 50 % that are far below empirical estimates found in the literature (∼80–90%). Conversely, we show that fixing *α*∼80–90% leads to a much tighter CI_95%_ for *ω*^−1^ that excludes 0. As such, we conclude that despite its broad CI_95%_ the window of susceptibility is justified for the sake of biological realism.

### Immunodynamics

**Fig. 3:**
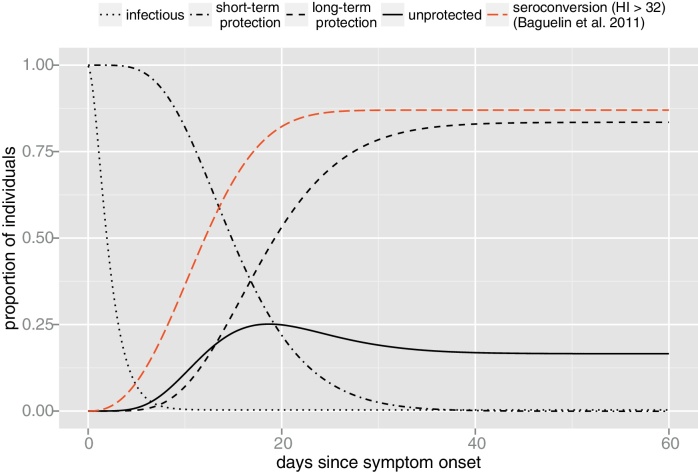
Dynamics of the immune response, under the SEICWH model, inferred from the 1971 TdC epidemic. At the population level, our framework allows us to reconstruct the proportion of individuals that are infectious (dotted black line), short-term protected thanks to the innate and cellular immunity (dot-dashed black line), protected on the long-term thanks to antibodies (dashed black line) and unprotected (solid black line) by interval since symptom onset. These proportions correspond, respectively, to the probabilities *P*_1–4_(*τ*) described in section “Quantities of epidemiological interest”. The dashed red line corresponds to the proportion of individuals seroconverted by interval since symptom onset obtained by Baguelin et al. (2011). This study involved 115 individuals infected during the 2009 A/H1N1 pandemic and the seroconversion interval of each individual was defined as the time taken to reach an HI titre of ≥32. (For interpretation of the references to color in this figure legend, the reader is referred to the web version of the article.)

The immunodynamics under our ML estimates is summarized in Fig. 3. In particular, we find that the waiting time in the window of susceptibility is exponentially distributed, thus revealing a high level of host heterogeneity in the development of the humoral response. Indeed, among those who do mount a protective humoral immune response (83% of the population), 30% will stay in the window of susceptibility less than one day and 20% more than four days (see Fig. S1, green curve). It also shows that, at the population level, the probability *P*_4_(*τ*) to sample an unprotected individual rapidly peaks to 0.25 three weeks after the date of symptom onset, owing to the window of susceptibility, and then decreases to (1 − *α*) = 0.17 on a longer time scale due to the lack of humoral protection.

Finally, we investigate the link between seroconversion and efficient protection by comparing the cumulative distribution obtained by Baguelin et al. (2011) with our probability distribution *P*_3_. Fig. 3 suggests that seroconversion occurs faster (∼1 week) and in a slightly greater proportion of infected hosts (87% vs. 83%) than efficient protection. We discuss this discrepancy in section “Immunodynamics model”.

### Model fit

In order to better characterize the 1971 two-wave epidemic on TdC, we simulated 10^5^ realizations of the stochastic SEICWH model (Fig. 1B) under the ML estimates (Table 2) and computed the 50 and 95 percentile intervals (PI_50,95%_) of the distribution conditioned on non extinction. Fig. 2 (upper panel) demonstrates the goodness of fit of the SEICWH model since the data lie in the PI_95%_ while the shape and dynamics of the epidemic are closely captured by the mean predicted incidence with PI_50%_ envelope (we refer to Text S8 for a similar analysis using parameter sets sampled from the CI_95%_).

In addition, Fig. 2 (lower panel) reveals that although the extinction probability increases at the beginning of the epidemic and during the inter-wave period (i.e. when the transmission chain can be broken due to the low number of infectious hosts), the risk of disease fadeout remains below 5%. By contrast, this risk rapidly increases during the downturn of the second epidemic wave due to depletion of the susceptible pool (i.e. most HR individuals have gained long-term protection). We can thus conclude that, despite the small community size of TdC, the infection and HR dynamics were robust to demographic stochasticity during the 1971 epidemic. Put another way, given the population settings of TdC, the HR wave was not a twist of fate but did have a high probability to occur.

### Interplay between the immunological and epidemiological dynamics

#### Three typical epidemic profiles

**Fig. 4:**
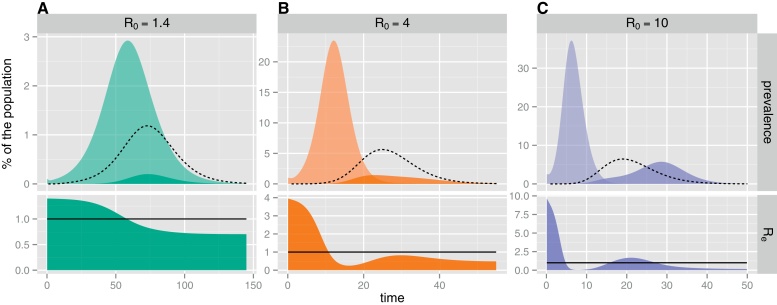
Example of the three typical epidemic profiles generated by the SEICWH model, depending on the value of *R*_0_. A: *R*_0_ = 1.4, B: *R*_0_ = 4 and C: *R*_0_ = 10. These values reflect the tendency of the contact rate among individuals to increase as the community becomes smaller. Upper panels: contribution of infection and HR to the time series of the prevalence. The dashed line represents the average daily inflow of unprotected individuals ($U_{d}$). Lower panels: time course of the effective reproduction number *R*_*e*_(*t*). The solid line represents the threshold *R*_*e*_ = 1, above which the epidemic can grow. (For interpretation of the references to color in text, the reader is referred to the web version of the article.)

The first striking result of the exploration is that three typical epidemic profiles can be distinguished, depending on the value of *R*_0_. When the contact rate is high (*R*_0_ ⪆ 5), as on TdC, the epidemic is composed of two waves with two distinct peaks (Fig. 4C). By contrast, at intermediate contact rates (*R*_0_ ∈ [2 − 5]), the epidemic is composed of a single wave with a “long tail” end (Fig. 4B). Finally, when the contact rate is low (*R*_0_ ⪅ 2), the tail disappears so that the epidemic becomes bell shaped (Fig. 4A). These three epidemic profiles arise from the interplay between the immunological and epidemiological dynamics that modulates the effective reproduction number *R*_*e*_(*t*) throughout the epidemic. For our model, *R*_*e*_(*t*) = *R*_0_(*S*(*t*) + *W*(*t*))/*Ω* and as long as *R*_*e*_(*t*) > 1 the epidemic is increasing.

In the parameter region of high *R*_0_ (Fig. 4C), the disease spreads so rapidly that almost the entire population is infected over a short time interval which is similar to the duration of the cellular protection. As a result, many recovered hosts lose their cellular protection simultaneously, leading to an important inflow of unprotected individuals $\left(U_{d}\right)$ shortly after the end of the infection wave. Accordingly, *R*_*e*_ rapidly increases so that, in the event that the chain of transmission is maintained until the threshold *R*_*e*_ = 1 is reached, HR becomes sustained and a second epidemic wave is observed.

By contrast, in the parameter region of low *R*_0_ (Fig. 4A), the disease spreads over a much longer time scale than the immune response so that $U_{d}$ peaks during the downturn of the infection wave. This timing, together with the low *R*_0_, help to explain why *R*_*e*_ remains below one after the first epidemic peak. In this case, the reinfection wave is not sustained but mainly driven by the infection wave.

Finally, in the parameter region of intermediate *R*_0_ (Fig. 4B), the disease spreads slowly enough that the reinfection wave is initially driven by the infection wave while $U_{d}$ is sufficient to maintain the chain of transmission after the end of the infection wave. However, in contrast to the high *R*_0_ case, *R*_*e*_ remains below one so that the epidemic does not peak again, but subsides in a tail of reinfection.

#### The HR threshold

**Fig. 5:**
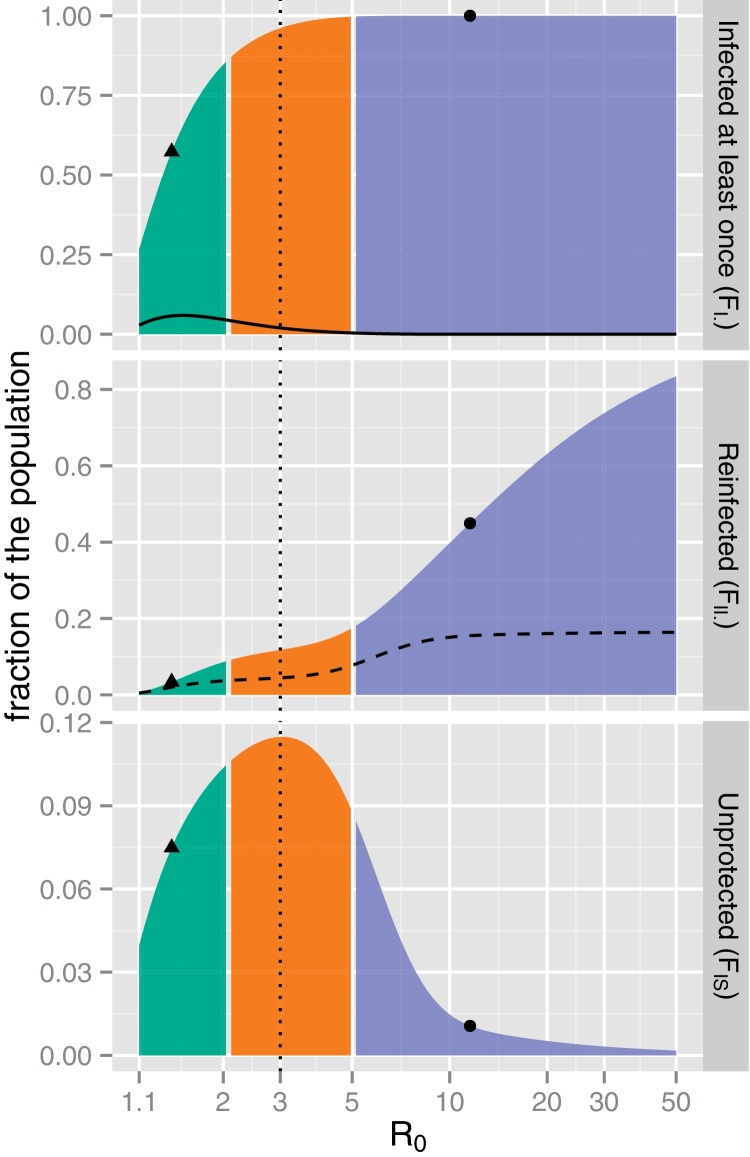
Change in the fractions of infected (*F*_*I*._, upper panel), reinfected (*F*_*II*._, middle panel) and unprotected (*F*_*IS*_, lower panel) individuals at the end of the epidemic as a function of *R*_0_. Each colour refers to an epidemic profile of Fig. 4 (bell: green, tail end: orange, two-peaks: violet). The impact of the reinfection dynamics on *F*_*I*._ can be obtained by subtracting the same fraction expected under the SEIR model (solid line, upper panel). The expected fraction of individuals reinfected following a lack of humoral response corresponds to *αF*_*I*._ − *F*_*IS*_ (dashed line, middle panel). The HR threshold $R_{0}^{*}$ is plotted as a dotted line and estimates of *R*_0_ for the 2009 A/H1N1 pandemic (≈1.4, black triangle) and for the 1971 TdC epidemic (≈12, black dot) are also mapped. Note the log-scale on the *x*-axis. (For interpretation of the references to color in this figure legend, the reader is referred to the web version of the article.)

As shown in Fig. 5 (upper panel), the fraction of individuals infected at least once during the epidemic (*F*_*I*._) increases rapidly with *R*_0_. In particular, the value of *F*_*I*._ is greater than expected with a SEIR model since the latter does not account for new cases resulting from contact with reinfected hosts (the difference is plotted as a dotted-line).

Regarding the fraction of the population that is reinfected (*F*_*II*._, middle panel) or that remains unprotected (*F*_*IS*_, lower panel) at the end of the epidemic, we note a qualitative change at intermediate values of *R*_0_. Specifically, we define the HR threshold by $R_{0}^{*}={\mathrm{argmax}}_{R_{0}}F_{\mathrm{IS}}(R_{0})$. When $R_{0}<R_{0}^{*}$, most hosts have closed their window of susceptibility before re-exposure to the virus whereas most of those with deficient humoral response are likely to escape HR until the end of the epidemic and remains unprotected. By contrast, when $R_{0}>R_{0}^{*}$, most hosts are likely to be re-exposed to the virus before their window of susceptibility closes whereas most unprotected hosts gain long-term immunity via HR during the second epidemic wave. We numerically calculated $R_{0}^{*}=3$ under the ML estimates of the immunological parameters.

Finally, we contend that this HR threshold should not be confused with the reinfection threshold introduced by Gomes et al. (2004). Although both thresholds indicate important qualitative change of the epidemic dynamics, we show in Text S9 that they have different epidemiological interpretations as well as different dynamical implications.

#### Implications for the first post-pandemic season

**Fig. 6:**
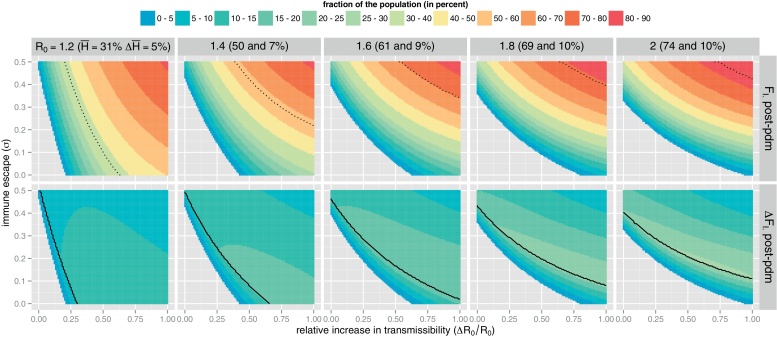
Implication of immunodynamics for the first post-pandemic season. Upper panels: expected fraction of individuals infected at least once by a mutant strain during the post-pandemic season (*F*_*I*._ post-pdm, colour coded), as a function of the relative increase in transmissibility (Δ*R*_0_/*R*_0_ ∈ [0, 1]) and the level of immune escape (*σ* ∈ [0, 0.5]) to the pandemic strain (the choice of these parameter ranges is justified in Text S5 based on published studies). Results are given for five reasonable values of *R*_0_ for pandemic scenarios (between 1.2 and 2). For each value of *R*_0_, the expected fraction of protected ($\bar{H}$) and unprotected ($\Delta \bar{H}$) individuals at the end of the pandemic are also given. The fraction $\bar{H}$ is therefore partially protected against the mutant strain by a factor of reduction of susceptibility 1 − *σ* (see also Text S5). Finally, the fraction of infected individuals during the pandemic season (*F*_*I*._ pdm) is also mapped as a black dotted isocline for comparison with *F*_*I*._ post-pdm (colour-coded). Lower panels: effect of assuming that all infected individuals develop an efficient humoral response during the pandemic and thus a partial-protection against the mutant strain. We compared the SEICWH model with initially $\bar{H}$ partially protected individuals with a SEIR model with $\bar{H}+\Delta \bar{H}$ as initial condition and plotted the difference (Δ*F*_*I*._ post-pdm, colour-coded). Since the SEIR model overestimates the population herd immunity, it predicts a greater invasion threshold for the post-pandemic mutant (isocline *R*_*e*_ = 1, black solid line) than the SEICWH model. (For interpretation of the references to color in this figure legend, the reader is referred to the web version of the article.)

Fig. 6 (upper panels) shows the expected fraction of individuals infected at least once by a mutant during the first post-pandemic season (*F*_*I*._ post-pdm) as a function of the immune escape *σ* and the relative increase in transmissibility Δ*R*_0_/*R*_0_, for five different values of *R*_0_ in agreement with pandemic scenarios in large populations (Lessler et al., 2007). As *R*_0_ increases the pandemic becomes more and more severe so that the expected fraction of protected individuals at the beginning of the post-pandemic season ($\bar{H}$) increases. Accordingly, from an evolutionary point of view, it becomes more and more efficient for the mutant to increase its immune escape than its transmissibility in order to invade the population. By contrast, when *R*_0_ is close to 1, a mutant antigenically similar to the pandemic strain can invade the population following moderate increase in transmissibility.

This pattern can be compared to that predicted by a SEIR model, i.e. assuming that all the individuals infected during the pandemic develop an efficient humoral response and are therefore partially protected against the mutant strain. One can show that, at the beginning of the post-pandemic season, the SEIR model underestimates the value of *R*_*e*_ for the mutant strain by $(R_{0}+\Delta R_{0})(1-\sigma )\Delta \bar{H}$, where $\Delta \bar{H}=F_{\mathrm{IS}}$ is the fraction of unprotected hosts at the end of pandemic season in the SEICWH model. Fig. 6 (lower panels) reveals a parameter region, below the invasion threshold (*R*_*e*_ = 1) of the SEIR model, where the SEICWH model predicts epidemics involving up to 25% of the population. Furthermore, even above this invasion threshold, the epidemic sizes differ by the same order of magnitude as a typical seasonal influenza epidemic (Δ*F*_*I*._ ≈ 5–20%).

## Discussion

### Immunodynamics model

Our study supports the view that host heterogeneity in the timely development of a protective immunity can explain HR. More precisely, although short lived (innate and cellular) immunity should prevent HR within 2–3 weeks following the primary infection (Cowling et al., 2012), incomplete immune formation and non seroconversion can lead to HR following re-exposure to the same strain on an intermediate and a long time-scale, respectively. These mechanisms provide an explanation to the HR cases reported over 2–5 weeks (Perez et al., 2010; Kim et al., 2010) as well as over several months (Trakulsrichai et al., 2012) during the 2009 A/H1N1 pandemic.

To our knowledge, the present statistical analysis is the first one that attempts to provide joint estimates for the duration of the short-term protection, the duration of the window of susceptibility and the probability to develop a long-term protection. Although strong correlations between *ω*^−1^ and *α* prevent us from identifying these key quantities with tight CI_95%_, our ML estimate of *α* is in very good agreement with empirical estimates in the literature. Moreover, we show in Text S7 that fixing *α* around these empirical estimates leads to much tighter CI_95%_ for *ω*^−1^. We have so far assumed that hosts are fully susceptibility to HR while in the window of susceptibility. In Text S10 we show that partial susceptibility can be modelled by means of an extra parameter and has the effect to lengthen the window of susceptibility. However, we also found that this extra parameter suffers from serious identifiability issue and choose not to include it in the present SEICWH model.

Finally, comparison of the immunological dynamics under the best fit model with empirical estimates from the 2009 pandemic in the UK (Fig. 3) suggests that either (i) seroconversion occurred more rapidly during the 2009 A/H1N1 pandemic in the UK than during the 1972 A/H3N2 epidemic on TdC or (ii) it should take a higher HI titre than 32 for efficient protection against HR. The first explanation could be justified by different immunogenic properties between A/H1N1 and A/H3N2 as well as different immuno-genetic background between the UK and TdC populations. However, we note that ML estimates of the immunological parameters for the TdC epidemic are in close agreement with empirical literature (see section “The primary immune response to influenza infection in humans”). Accordingly, we believe that our estimates can reasonably be extended to other human populations and influenza viruses. On the other hand, the second explanation is in good agreement with the results of a recent meta-analysis showing that a HI titre of 32 corresponds to less than 50% reduction in the risk of contracting influenza whereas it takes a titre of ≥100 to decrease this risk to 10% (Coudeville et al., 2010). Similarly, we note that most labs outside UK fix the protective threshold at 40 instead of 32, thus increasing the time to seroconversion while reducing the proportion of seroconverted.

### Does HR drive multiple-wave influenza outbreaks?

Our study indicates that HR could drive multiple-wave influenza outbreaks in communities with exceptional contact configurations like schools or isolated settlements. However, we have assumed so far a constant contact rate between infected and susceptible (or unprotected) individuals as well as no prior immunity to the new virus. These assumptions seem justified for the isolated and close-knit TdC community. Indeed, the high attack rate (96%) during the 1971 epidemic (Mantle and Tyrrell, 1973) suggests that those who were infected at the beginning of the infection wave have rapidly been re-exposed while caring for the sick during the inter-wave period, thus initiating the HR wave. By contrast, we expect that in less isolated and better prepared communities, past influenza exposures and vaccination should reduce the number of susceptible individuals at the beginning of the epidemic, thus increasing the HR threshold $R_{0}^{*}$. On the other hand, distancing or containment measures should rapidly be implemented as the epidemic progresses, thus considerably mitigating the risk of re-exposure. For instance, school closure could rapidly drive the epidemic to extinction, whereas rapid isolation of suspected cases could efficiently reduce the contact rate of infected host, thus preventing the HR wave.

In line with these scenarios, no reinfection was reported during an influenza outbreak that occurred in a boarding school of 763 boys in 1978 (one year after the re-emergence of A/H1N1) despite the high attack rate (67%) (Anonymous, 1978). In this case, infectious boys were confined to bed and cared by 130 adults, presumably already immune as only one of them reported symptoms. As a result, the epidemic died out after 13 days, while most of the recovered boys would still have benefited from a cellular protection (see Fig. S11). By contrast, in 1924, only 121 (13%) of 904 boys of the Royal Navy School of Greenwich had already been infected after 23 days of disease propagation (presumably because of the high level of prior-immunity acquired since the 1918 pandemic), when 40 new boys were distributed indiscriminately throughout the school (Dudley, 1926). On the 26th day, two of this batch developed influenza, and within a week nine new boys had been infected. Meanwhile the incidence among the old boys, which had previously been on the wane, rose again, and 12 reinfections were reported. Interestingly, a further batch of 40 new boys were introduced just at the end of the epidemic, when the chances of infection must have considerably diminished; six of these were ultimately infected during the period in which 14 cases of infection and 4 cases of reinfection were reported among the old boys (see Fig. S12). This epidemic pattern can simply be explained by an increase of *R*_*e*_ due to the simultaneous effect of the replenishment of the susceptible pool and the inflow of unprotected old boys.

On the other hand, our results clearly indicate that HR is not sufficient in itself to generate the multiple-wave outbreak patterns observed during past pandemics in large populations. Indeed, *R*_0_ has been estimated around 2 (Lessler et al., 2007) which is below the HR threshold $R_{0}^{*}$. In these cases, HR would have only increased the force of infection, and thus the number of infected hosts, by a few percent. Once again, we contend that our simple transmission model willingly ignores many known mechanisms at work in larger and more structured populations such as age-dependency in the contact rate (Mossong et al., 2008) (i.e. heterogeneous mixing) and behavioural changes (Funk et al., 2009). Furthermore, propagation of a new influenza virus over several months must depend on seasonal variations in transmissibility (i.e. change in absolute humidity (Shaman and Kohn, 2009)) and contact rate (i.e. school closing and reopening (Hens et al., 2009)) as well as meta-population coupling (Balcan et al., 2009). Indeed, recent studies suggest that the timing of the first and second waves during the 2009 pandemic influenza was controlled by a combination of these mechanisms (Chao et al., 2010; Shaman et al., 2010). Nevertheless, despite the simplicity of our transmission model, we believe that our qualitative conclusions on the role of HR in large populations (i.e. below the HR threshold) remain valid even including these additional mechanisms.

### The necessity to account for immunodynamics and host heterogeneity in epidemiological models

In communities with exceptional contact rates, HR can rapidly drive a second epidemic wave involving not only individuals with deficient humoral response but also those who are re-exposed before their humoral protection becomes efficient. Accordingly, the risk of a wave of reinfection cannot be anticipated without a precise description of the immunodynamics that follows recovery from influenza infection as in the SEICWH model. By contrast, in larger and less mixed populations, HR does not significantly alter the epidemiological dynamics so that a simple SEIR framework should be sufficient to predict or infer (retrospectively) the impact of a new virus in these populations.

On the other hand, one should also bear in mind that the SEIR model overestimates the level of population immunity at the end of the pandemic by assuming that all infected hosts develop a protective humoral response. Accordingly, our results indicate that consideration of host heterogeneity in the humoral response is essential in order to anticipate the impact of a mutant in the post-pandemic era. In addition, this would permit to quantify the fraction of infected hosts that should remain unprotected after a pandemic and could therefore benefit from vaccination in order to boost their humoral response. Although it seems difficult to separate protected from unprotected hosts without individual serological tests, we can nonetheless assume that random vaccination of symptomatic cases should, in principle, increase population herd immunity through cross-immunity to subsequent antigenic variants.

The SEICWH model represents a step forward in the consideration of the immune response, and its heterogeneity among individuals, in epidemiological models. However, further research and refinements could be envisaged to improve its realism. First, reinfected hosts may benefit from T-cell “memory” and be less infectious than infected hosts, and even more often asymptomatic, thus reducing their risk to transmit the disease. Second, host heterogeneity in the development of a protective humoral response could vary depending on the immunogenic properties of each influenza virus and the population under study. For instance, it was recently reported that although 90% of the infected hosts in the age range 16–29 seroconverted during the 2009 pandemic, this proportion decreased to 70% for those aged 50 years and over (Hung et al., 2010). Finally, although we have assumed a life-long humoral protection once in the *H* stage, the same study conducted during the 2009 pandemic also revealed that 7 and 16% of patients who seroconverted had a decline of antibody titre of 4- and 2-fold, respectively, after one year (Hung et al., 2010). As for the lack of immune response, this additional mechanism could have significant implications for the current post-pandemic era by increasing the effective reproduction number of subsequent nH1N1 variants.

## Figures and Tables

**Table 1:** Transitions between classes in the stochastic SEICWH model. The notation *A* → *B* means that when the event occurs one individual is transferred from compartment *A* to compartment *B*.

Event	Transition	Rate at which event occurs
(re)Infection	*S* → *E*_1_	*βs*(*i*_1_ + *i*_2_)/*Ω*
	*W* → *E*_1_	$\beta w(i_{1}+i_{2})/\Omega$
Progression of incubation	*E*_1_ → *E*_2_	2*ϵe*_1_
Start of infectiosity	*E*_2_ → *I*_1_	2*ϵe*_2_
Progression of infectiosity	*I*_1_ → *I*_2_	2*νi*_1_
Recovery	*I*_2_ → *C*_1_	2*νi*_2_
Progressive loss of cellular protection	*C*_*k*_ → *C*_*k*+1_	5*γc*_*k*_
Deficiency of humoral response	*C*_5_ → *S*	(1 − *α*)5*γc*_5_
Start of the window of susceptibility	*C*_5_ → *W*	*α*5*γc*_5_
Start of the humoral protection	*W* → *H*	ωw

**Table 2:** Results of the maximum likelihood statistical inference for the 1971 TdC epidemic. ML estimates and 95% confidence intervals for the SEICWH model parameters.

Symbol	Description	Estimate	CI_95%_
*R*_0_ = *β*/*ν*	Basic reproduction number	11.78	7.70–25.50
1/*ϵ*	Mean latent period (days)	2.18	1.53–2.96
1/*ν*	mean infectious period (days)	2.32	0.70–5.03
1/*γ*	Mean temporary removed period (days)	13.37	10.37–16.31
1/*ω*	Mean duration of the reinfection window (days)	2.75	0–6.03
*α*	Probability to develop long-term immunity	0.83	0.49–1
*ρ*	Reporting rate for observation	0.71	0.62–0.82
*I*_0_	Number of initially infectious individuals	1	1–3
*S*_0_	Number of initially susceptible individuals	277	275–280

*l*(*θ*_ML_)	Maximized log-likelihood	−112.19	–
