# Supplementary material for: Does homologous reinfection drive multiple-wave influenza outbreaks? Accounting for immunodynamics in epidemiological models
Source: Epidemics. 2013 Dec;5(4):187–96. doi: 10.1016/j.epidem.2013.09.003 (PMC3863957; doi:10.1016/j.epidem.2013.09.003)
Supplement: Supplementary file 1 [file mmc1.pdf]

Does homologous reinfection drive multiple-wave influenza outbreaks? Accounting for immunodynamics in epidemiological models Electronic supplementary material

Anton Camacho[1,2,*], Bernard Cazelles[1,3]

1 Eco-Evolution Mathématique, UMR 7625, CNRS-UPMC-ENS, 75230 Paris Cedex 05, France
2 Department of Infectious Disease Epidemiology, London School of Hygiene and Tropical Medicine, London, United Kingdom
3 UMMISCO UMI 209 IRD-UPMC, F-93142 Bondy, France

* *Corresponding author*:
E-mail: anton.camacho@lshtm.ac.uk

Contents

1 Text S1: The humoral immune response to influenza in humans **2**

2 Text S2: Use of Erlang distributions in the SEICWH model **2**

3 Text S3: Deterministic approximation of the stochastic SEICWH model **4**

4 Text S4: Historical version of the SEICWH model **4**

5 Text S5: The SEICWH model for the post-pandemic period **6**

6 Text S6: Pre-existing immunity to influenza A/H3N2 on TdC before 1971 **6**

7 Text S7: Discussion on the broad confidence intervals for ω^{-1} **7**

8 Text S8: Assessment of model fit from the $CI_{95\%}$ **7**

9 Text S9: Comparison between the HR threshold and the reinfection threshold of Gomes et al. [1] **9**

10 Text S10: Partial susceptibility in the W stage **10**

11 Text S11: Historical data **11**

1 Text S1: The humoral immune response to influenza in humans

It is known that mucosal antibodies (Abs) – mainly IgA – protect the upper respiratory tract (URT) whereas serum Ab – mainly IgG – protect the lower respiratory tract (LRT) [2]. Most influenza infections occur through small airborne particles that deposit in the LRT [3]. As such, it has been reported that serum Abs correlate better than mucosal Abs with protection though it depends on the infection route (LRT vs URT) [3].

That said, we contend that the distinction between mucosal and serum responses is not necessary for our study as both responses share the essential properties that form the basis of the reinfection mechanisms accounted by the SEICWH model:

- their inherent host heterogeneity: for instance Tamura et al. report on page 239 of [4]: "Approximately 85% of seronegative adult volunteers who are infected with a live attenuated virus developed an IgA Ab response". Similarly, it has been reported that a protective serum antibody response cannot be detected in approximately 10 to 20% of subjects after natural influenza infection [5, 6].
- the time-scale of their dynamics: both responses peak after completion of the cellular response, several weeks following infection [7, 3, 8].
- both IgA and IgG remain detectable for several years after infection [3, 5].

As such, in the present study, we can reasonably consider the humoral response in general without distinguishing further between mucosal and serum Ab. Indeed, the timing of the window of susceptibility remains in good agreement with the interval between the completion of CTL contraction and the full development of the humoral (mucosal and serum) response.

2 Text S2: Use of Erlang distributions in the SEICWH model

The flexibility of the Erlang distribution ranges from the exponential ($k = 1$) to Gaussian-like ($k >> 1$) distributions. This qualitative change is illustrated in Figure S1. In particular, note that the density in zero, which is always positive for the exponential distribution, becomes zero when $k > 1$. In the main paper we obtained different values for the mean and shape parameters for the distributions of the duration in the successive immunological stages. In the Figure S2 we plot the probability density function (PDF) and the cumulated density function (CDF) of each duration, together with their $\text{CI}_{95\%}$ bounds.

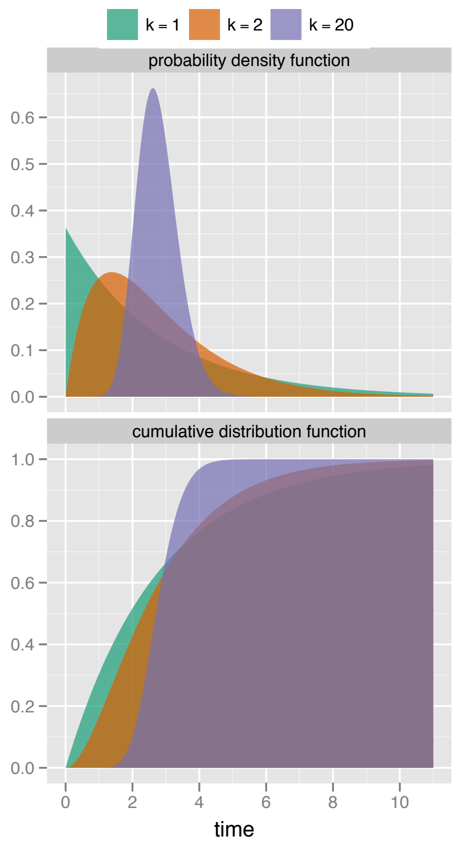

Figure S1: Gamma distributions with mean duration $m = 2.75$ days (corresponding to the mean duration of the window of susceptibility inferred from the TdC data set) and shape $k = 1, 2$ and 20 (colour coded). The case $k = 1$ corresponds to the exponential distribution.

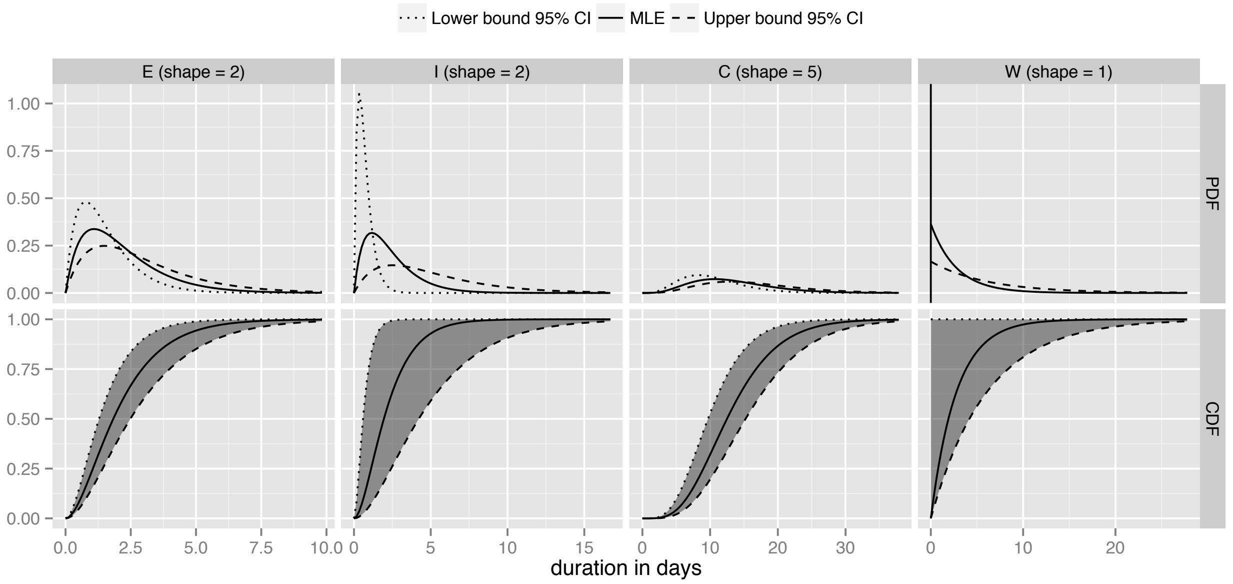

Figure S2: Probability density function (PDF, upper panels) and cumulated density function (CDF, lower panels) of the duration in the successive immunological stages (E,I,C and W). The solid black line corresponds to the ML estimate and we also plotted the curves corresponding to the lower (dotted) and upper (dashed) bounds of their $CI_{95\%}$.

3 Text S3: Deterministic approximation of the stochastic SEICWH model

In the SEICWH model, the main stochastic effect is the risk of epidemic fadeout during the trough between epidemic waves. As such, the use of a deterministic approximation is only justified when the probability of stochastic extinction remains negligible for the range of parameter values explored ($R_0 > 1$ and $\Omega > 1000$).

First, we show in Figure 4 (main paper) that the two distinct waves only occur when $R_0 > 5$. Therefore, demographic stochasticity is not expected to play a major role when $R_0 < 5$ and, in this case, the deterministic model provides an accurate description of the average epidemic dynamics.

Second, when $R_0 > 5$, we argue that the deterministic model still captures the average dynamics for the parameter values of interest. We recall that the deterministic model is the large population limit of the stochastic model in the sense that, for a fixed time t, all other parameters being equal, one can always find a population size Ω large enough so that the stochastic dynamic can be approximated by the deterministic one. In our context, this means that one can always find a population size large enough so that the risk of stochastic extinction during the epidemic trough is arbitrarily small. We thus used the stochastic SEICWH model to compute the extinction probability when $R_0 = 5.5$ (i.e. beginning of the two-wave dynamics) for $\Omega = 284$ (i.e. the TdC population size) and $\Omega = 1000$ and obtained values below 10^{-1} and below 10^{-3} respectively. Since this probability decreases as R_0 and/or Ω increase, we can conclude that even in the two-wave case, the risk of stochastic extinction becomes negligible as the population size exceeds that of TdC. This justifies the use of deterministic simulations for the range of parameter values explored ($R_0 > 1$ and $\Omega > 1000$) in the rest of the paper.

The set of ODEs for the deterministic SEICWH model:

$$\dot{S}_1 = -\beta S_1 \frac{I_1 + I_2}{\Omega} - 5\gamma(\alpha - 1)C_5 \quad (1)$$

$$\dot{E}_1 = \beta(S_1 + W)\frac{I_1 + I_2}{\Omega} - 2\epsilon E_1 \quad (2)$$

$$\dot{E}_2 = 2\epsilon(E_1 - E_2) \quad (3)$$

$$\dot{I}_1 = 2\epsilon E_2 - 2\nu I_1 \quad (4)$$

$$\dot{I}_2 = 2\nu(I_1 - I_2) \quad (5)$$

$$\dot{C}_1 = 2\nu I_2 - 5\gamma C_1 \quad (6)$$

$$\dot{C}_2 = 5\gamma(C_1 - C_2) \quad (7)$$

$$\dot{C}_3 = 5\gamma(C_2 - C_3) \quad (8)$$

$$\dot{C}_4 = 5\gamma(C_3 - C_4) \quad (9)$$

$$\dot{C}_5 = 5\gamma(C_4 - C_5) \quad (10)$$

$$\dot{W} = 5\gamma\alpha C_5 - W(\beta(I_1 + I_2) + \tau) \quad (11)$$

$$\dot{H}_1 = \tau W \quad (12)$$

4 Text S4: Historical version of the SEICWH model

In order to track both the infection and the immunological histories of individuals it is necessary to use a different version of the SEICWH model than the one presented in Figure 1B of the main paper. In the historical version, the immunological stages have a superscript that corresponds to the number of (re)infections. For instance, individuals who have been infected only once without developing an efficient humoral response remain in the immunological stage S^1 whereas those who have been reinfected and have developed an efficient humoral response are in the immunological stage H^2. Mathematically, the original version of the SEICWH is equivalent to the historical version presented in Figure S3 which has a dimension equal to $12n$, where n is the maximal number of reinfection episodes. In the original model, individuals can be reinfected an unlimited number of times (for instance when $\alpha = 0$) so that, rigorously, the historical version is not tractable ($n \to \infty$). However, since we are mostly interested in the first reinfection event, we choose to keep the historical model tractable by allowing only one reinfection episode. This simplification can be justified by two observations. First, we fitted the original model (i.e. with no upper bound for the number of reinfections) on the TdC data set and obtained $\alpha = 0.83$. Accordingly, the expected chance to remain unprotected after the first reinfection episode is less than 3% so we can reasonably neglect subsequent reinfection. Second, individuals who have a delayed humoral response and are reinfected before their window of susceptibility closes are expected to develop humoral immunity more rapidly than following primary infection and are thus unlikely to pass again through a W^2 stage, which can be omitted. These two assumptions allow us to obtain the historical version of the SEICWH model of Figure S4 (note that the $C^2_{1:5}$ stages are irrelevant in this model). In the model of Figure S4, the fraction of individuals that have been infected at least once during the epidemic is given by: $F_{I.} = (H^1 + S^1 + H^2)/\Omega$, the faction reinfected: $F_{II.} = H^2/\Omega$, and the fraction infected only once but that remains unprotected at the end of the epidemic: $F_{IS} = S^1/\Omega$. In the main paper, we used the deterministic version of the historical model in order to explore the reinfection dynamics in different population settings and to compute the quantities of epidemiological interest defined in section .

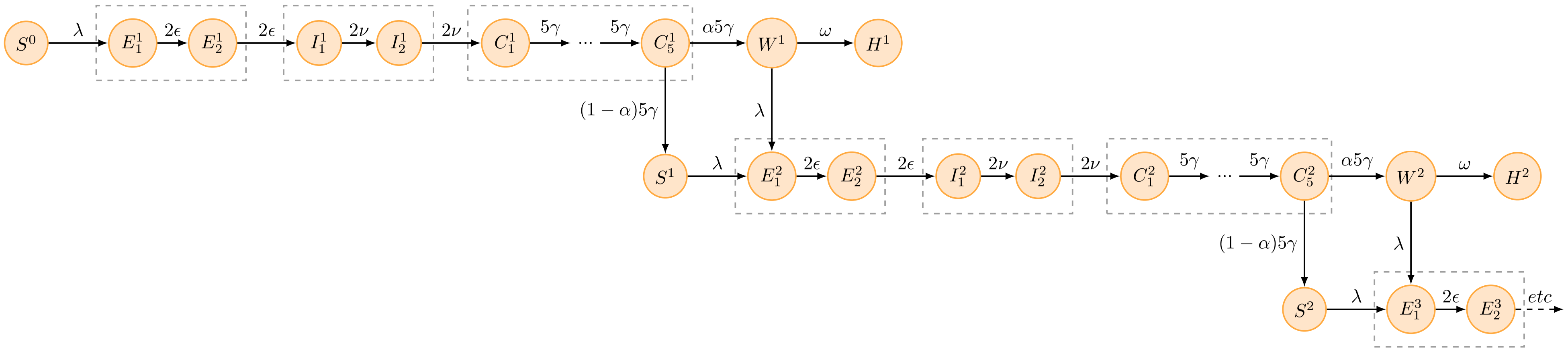

Figure S3: Historical version of the SEICWH model with infinite dimension. Parameters are described in Table 2 of the main paper. The force of infection is $\lambda = \beta \sum_{n=1}^{\infty}(I_1^n + I_2^n)/\Omega$

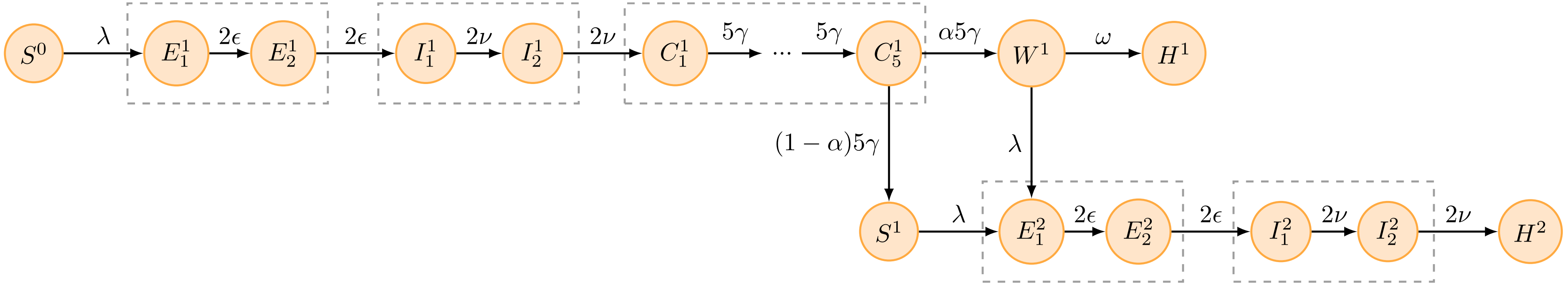

Figure S4: Historical version of the SEICWH model allowing for only one reinfection episode. Parameters are described in Table 2 of the main paper. The force of infection is $\lambda = \beta \sum_{n=1}^{2}(I_1^n + I_2^n)/\Omega$

5 Text S5: The SEICWH model for the post-pandemic period

During the post-pandemic period it is important to separate the individuals who have developed neutralizing antibodies against the pandemic strain (H^1) from those who escaped infection or remained unprotected at the end of the pandemic. Indeed, whereas the former are partially protected against strains that are cross-related to the pandemic one, the latter are fully susceptible to infection. More precisely, partially protected individuals benefit from a factor of reduction of susceptibility $1-\sigma$ i.e. they can be infected with probability σ following exposure. The post-pandemic SEICWH model is displayed in Figure S5.

On the other hand, we have assumed in the main paper that rapid evolution of the pandemic strain through mutations mainly results in changes of its antigenic properties (via σ) and/or its relative transmissibility (via $\Delta R_0/R_0$). From a practical point of view, it is difficult to predict the amount of changes, in terms of transmissibility and immune escape, between pandemic and post-pandemic strains. Nevertheless, previous studies that aimed to compare pandemic strains to their subsequent seasonal variants can give some valuable benchmarks. For instance, Fraser et al. [9] have recently estimated that the susceptible-infectious transmission probability on household was about twice greater for seasonal strains than for pandemic strains (corresponding to $0 \leq \Delta R_0/R_0 \leq 1$). Although the authors argue that caution is needed in drawing strong conclusions because of differences in the study design (for instance the seasonal studies focused on households with children), their results suggest that compensatory mutations that increase transmissibility might rapidly be selected as the new virus spreads in the human population. On another hand, the antigenic map of A/H3N2 main antigen (the hemagglutinin H3) by Smith et al. [10] shows that the first significant antigenic change occurred in 1972, four years after the pandemic caused by this new subtype. Results from modelling studies suggest that such an antigenic jump should correspond to an immune escape between 20 and 50% [11]. By contrast, results from a classical human experimental infection study following vaccine immunization [12] suggest a value between 0 and 20% [13]. In the main paper, we have assumed that $0 \leq \sigma \leq 0.5$.

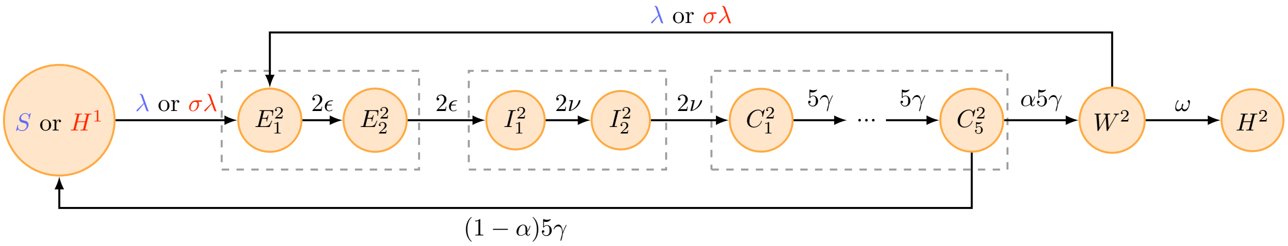

Figure S5: The flow of susceptible (partially protected) individuals at the beginning of the post-pandemic season corresponds to the blue (red) colour. In particular, partially protected individuals benefit from a factor of reduction of susceptibility $1-\sigma$ i.e. they can be infected with probability σ following exposure. More precisely, $\sigma \in [0,1]$ and $\sigma = 0$ means that the pandemic and post-pandemic strains are antigenically identical. The force of infection is $\lambda = \beta(I_1^2 + I_2^2)/\Omega$, where the superscript 2 indicates post-pandemic strain.

6 Text S6: Pre-existing immunity to influenza A/H3N2 on TdC before 1971

In the main paper we found that about 5 islanders (2% of the TdC population) would had already been immune and protected at the beginning of the 1971 epidemic. One potential explanation is that they were infected by the A/H3N2 virus before 1971 without sparking an epidemic on TdC. Indeed, the A/H3N2 virus emerged in 1968, thus leaving 3 years for the 5 islanders to get in contact with the virus, for instance during a trip on the continent. On the other hand, it can be shown (see the page 17 of the supplementary material of [14] for instance) that, in the SEICWH model, the probability that an epidemic dies out before a major outbreak is equal to

$$P = \left[\frac{1}{2}\left(1 + \frac{4}{R_0} - \sqrt{1 + \frac{8}{R_0}}\right)\right]^{I_0}.$$

Assuming $I_0 = 1$ and $R_0 > 10$ we obtain $P < 0.03$ and it is therefore unlikely that one initially infected individual recovers without sparking a major outbreak on such a close knit community. Thus, although some islanders could have been infected by the A/H3N2 virus before the 1971 epidemic, we speculate that these infections occurred outside of TdC, e.g. when travelling on the continent. We note however that no information is available on the frequency of these travels for the period 1968-1971.

7 Text S7: Discussion on the broad confidence intervals for ω^{-1}

The 95% confidence interval ($\mathrm{CI}_{95\%}$) for the expected duration of the window of susceptibility (ω^{-1}) includes 0. This suggests a more parsimonious model (SEICH) where all homologous reinfections (HR) are explained by a lack of protective humoral response in a proportion $1-\alpha$ of the infected individuals. However, it is important to emphasize that, as one fixes $\omega^{-1}=0$, and reduce the SEICWH model to the SEICH model, the maximum likelihood estimates (MLE) of the remaining parameters also change: in particular the MLE of α decreases to 53%. This is due to a strong correlation between α and ω in the SEICWH model: as the window of susceptibility shortens less individuals are reinfected so that the probability to develop a long-term protection must also decrease in order to fit the data.

Actually, in a previous study [14], we fitted the SEICH model to the TdC epidemic - using the same inference framework as in the present study - and we found that $\alpha_{MLE}=0.53$ with $\mathrm{CI}_{95\%}:[0.37-0.60]$. Accordingly, the SEICH model with $\alpha\sim 0.8$ (as in the SEICWH model) has a very low log-likelihood ($ll<-120$) and is rejected by likelihood ratio test ($p_{value}<10^{-4}$) in favour of the SEICWH model.

Finally, we note that even the upper bound of the $\mathrm{CI}_{95\%}$ of $\alpha(=0.60)$ for the SEICH model is far below the empirical estimates that can be found in the literature where α has been estimated between 0.8 and 0.9 [3, 6]. By contrast, it is remarkable that despite its wide $\mathrm{CI}_{95\%}$ ([0.49 - 1]), the MLE of α (=0.83) for the SEICWH model is in very good agreement with empirical estimates. Accordingly, we could have fixed α, based on the literature, and would have estimated a similar duration of the window of susceptibility as in the main paper but with a much tighter confidence intervals that excludes 0. Indeed, the log-likelihood profile shown in Figure S6 demonstrates that fixing $\alpha=0.8$ leads to $\omega^{-1}_{MLE}=2.10$ days, $\mathrm{CI}_{95\%}:[1.25-4.9]$. It is also clear that fixing α to higher values would result in greater estimates of ω^{-1}.

In summary, although we admit that the data are not sufficient to identify all model parameters with tight $\mathrm{CI}_{95\%}$, we contend that the two reinfection mechanisms should be accounted for in the SEICWH model for the sake of immunological realism. Although we could have overcome the issue of wide confidence intervals by fixing α using available empirical literature, we instead chose a more transparent approach which, together with appropriate explanations, make explicit the limitations of our data.

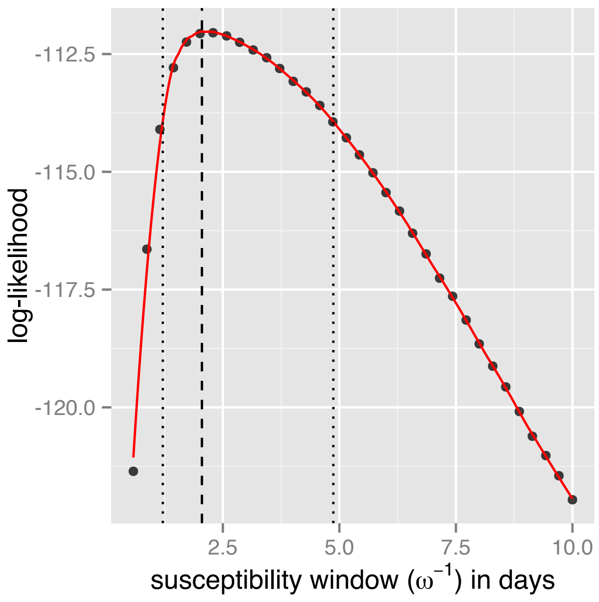

Figure S6: Log-likelihood profile for the susceptibility window (ω^{-1}) when the probability to develop a long-term protection (α) is equal to 0.8. We obtain $\omega^{-1}_{MLE}=2.10, \mathrm{CI}_{95\%}:[1.25-4.9]$ days

8 Text S8: Assessment of model fit from the $\mathrm{CI}_{95\%}$

In the main paper we have assessed model's fit under the MLE (Figure 2). Here we aim at comparing simulated epidemics when parameter sets are drawn from the $\mathrm{CI}_{95\%}$ distribution. Although we provide $\mathrm{CI}_{95\%}$ for each of the 10 parameters (Table 2, main paper) we cannot draw from these independently as we must account for correlations between parameters. This can easily be done in a Bayesian framework as one has access to the posterior distribution. By contrast, in the frequentist approach, this would require to evaluate the 10-dimensional likelihood surface around the MLE. When the likelihood is intractable, as in our case, this procedure is computationally too expensive.

Here, we propose to use the results of the 1-dimensional log-likelihood profiles already computed to obtain the $\mathrm{CI}_{95\%}$ of Table 2 (main paper). Indeed, since log-likelihood profiles maximize over all the other parameters, they account for

parameter correlations. Our idea is thus to compare the simulated epidemics under the MLE with those obtained under appropriate points of the 1-D profile. As a limit case, we choose θ_{low} and θ_{up} corresponding, respectively, to the lower and upper bounds of the $\text{CI}_{95\%}$. As a example, we use the log-likelihood profile of ω^{-1} (see Table S1 for the corresponding values of θ_{low} and θ_{up}).

Figure S7 (upper panels) shows that epidemics simulated under θ_{MLE}, θ_{low} and θ_{up} largely overlap due to demographic stochasticity. Indeed, the small population of TdC creates such variability over repeated simulations that the epidemics conditioned on having two waves appear similar for the three parameter sets. However, focusing on the extinction probability (Figure S7, lower panels) it becomes clear that the best model (θ_{MLE}) minimizes the extinction probability during the inter-wave period (so that the second wave is more likely) while maximizing this probability at the end of the second wave, thus preventing a third wave to occur. Accordingly, θ_{MLE} is the most likely parameter set.

At this point, it must be noted that θ_{low} and θ_{up} do not belong to the boundary of the $\text{CI}_{95\%}$ of the full parameter set. This is because the definition of the $\text{CI}_{95\%}$ explicitly depends on the dimension of the log-likelihood profile. More precisely, for a parameter vector θ of dimension d, if θ is partitioned into two components θ_1 and θ_2, of dimensions d_1 and d_2 respectively, then the log-likelihood profile of θ_1 is defined by:

$$l_p(\theta_1) = \sup_{\theta_2} l(\theta_1, \theta_2), \tag{13}$$

and an approximate $\text{CI}_{95\%}$ for θ_1 is given by:

$$\{\theta_1 : 2[l_p(\hat{\theta}_1) - l_p(\theta_1)] < \chi^2_{0.95}(d_1)\}, \tag{14}$$

where $\chi^2_{0.95}(d_1)$ is the 0.95 quantile of a χ^2 random variable on d_1 degrees of freedom, and $\hat{\theta}_1 = \text{argmax}\ l_p(\theta_1)$. Log-likelihood profiles are usually performed with $d_1 = 1$ or 2 and one can see from (14) that this procedure becomes computationally too intensive in the limit case where we want to obtain the $\text{CI}_{95\%}$ of the full parameter vector θ ($d = 10$). Indeed, this would require to evaluate $l_p(\theta_1)$ along an unknown 10-dimensional hyper-surface.

Given the likelihood of θ_{low} and θ_{up} (see Table S1) it can be shown, using (14), that these points belong to the $\text{CI}_{99.99\%}$ of θ. Alternatively, one can see from (14) that $\theta_{0.95}$ belongs to the boundary of the $\text{CI}_{95\%}$ of θ if and only if

$$2[l_p(\theta_{MLE}) - l_p(\theta_{0.95})] = \chi^2_{0.95}(10). \tag{15}$$

Using the log-likelihood profile of ω^{-1} we found that $\theta^*_{0.95}$, defined in Table S1, verifies the equality (15). More precisely, $\theta^*_{0.95}$ corresponds to $\omega^{-1} = 14$ days and can be seen as the upper bound of the $\text{CI}_{95\%}$ of θ in the direction of increasing ω^{-1}. The Figure S8 shows that, under $\theta^*_{0.95}$, the dynamics of the SEICWH model poorly captures the shape of the second epidemic wave (upper panel) and has a significant probability (~ 0.5) to fade-out during the inter-wave period (lower panel).

Overall, this analysis emphasizes the role of demographic stochasticity in the spread of influenza H3N2 in the small community of TdC and the need to account for this effect when fitting a dynamical model to the 1972 two-wave epidemic. Looking more closely at these stochastic effects, and at the extinction probability in particular, qualitative and quantitative differences appear among parameter sets from the $\text{CI}_{95\%}$.

Table S1: Parameter values used in the Figures S7 and S8. θ_{low} and θ_{up} correspond to the lower and upper bounds of the $\text{CI}_{95\%}$ of ω^{-1} whereas $\theta^*_{0.95}$ belongs to the $\text{CI}_{95\%}$ of the full parameter set θ.

Symbol	Description	θ_{low}	θ_{up}	$\theta^*_{0.95}$
$R_0 = \beta/\nu$	basic reproduction number	11.20	9.23	5.39
$1/\epsilon$	mean latent period (days)	2.07	2.02	1.88
$1/\nu$	mean infectious period (days)	2.44	2.15	0.97
$1/\gamma$	mean temporary removed period (days)	11.57	13.95	14.75
$1/\omega$	mean duration of the reinfection window (days)	0	6 .03	14.00
α	probability to develop long-term immunity	0.53	0.95	0.88
ρ	reporting rate for observation	0.71	0.68	0.66
I_0	number of initially infectious individuals	1	1	1
S_0	number of initially susceptible individuals	277	277	277
$l(\theta_{low,up})$	maximized log-likelihood	-112.78	-114.02	-121.34

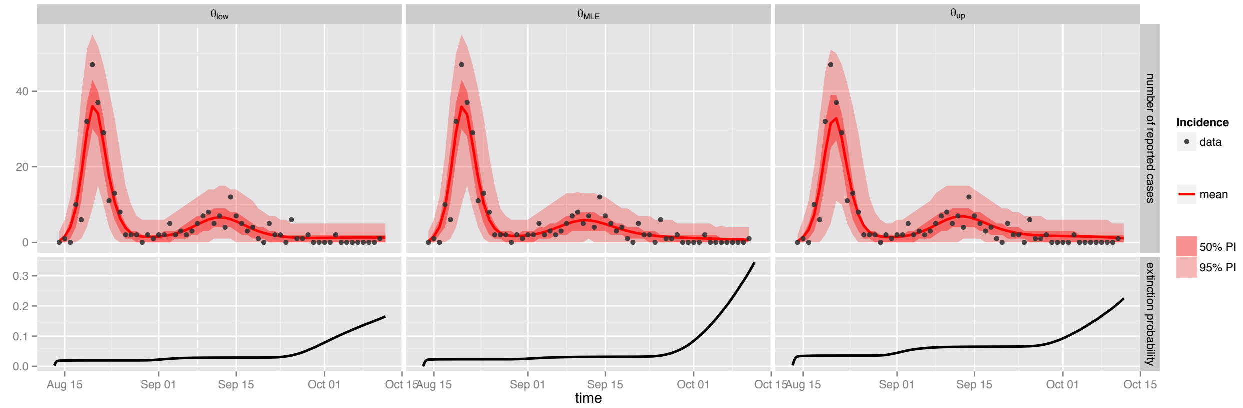

Figure S7: Comparison of simulated epidemics under three different parameter sets: θ_{MLE} is the best fit whereas θ_{low} and θ_{up} correspond to the lower and upper bounds of the $\mathrm{CI}_{95\%}$ of ω^{-1}. Upper panels: original incidence data (black dots) and expected incidence (red line) conditioned on non-extinction together with 50 and 95 percentiles (red envelopes) due to demographic stochasticity. Lower panel: time course of the extinction probability $p(t)$ defined as the probability that the epidemic has faded out by time t and estimated by the proportion of fade-out trajectories at time t. This figure reveals that the best fit of the SEICWH model should minimize the extinction probability during the inter-wave period (so that the second wave can occur) while maximizing this probability at the end of the second wave, in order to prevent occurrence of a third wave

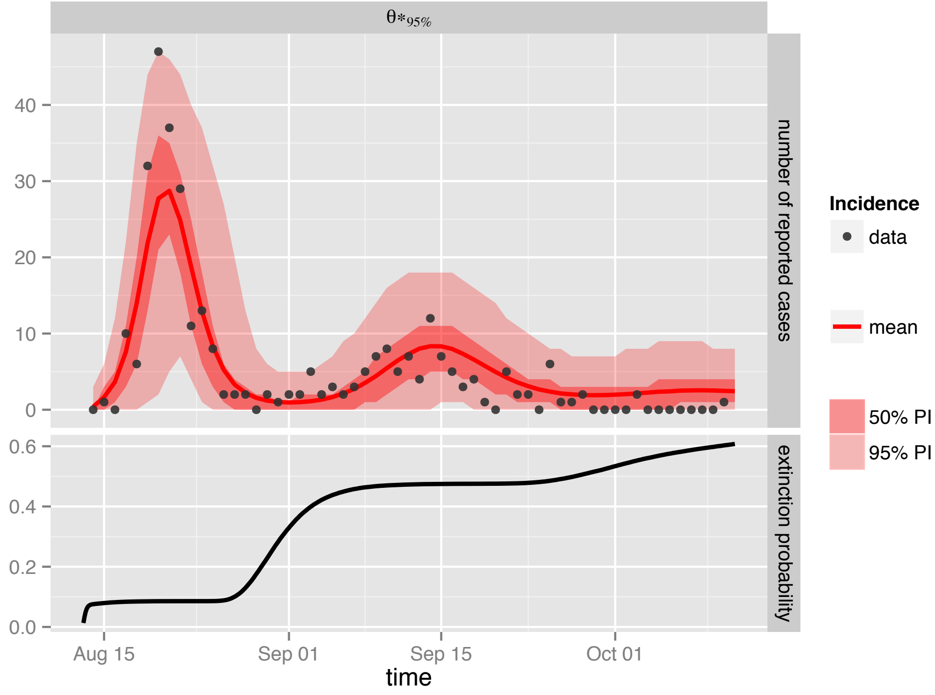

Figure S8: Simulated epidemics under the parameter set $\theta^*_{0.95}$, which belongs to the $\mathrm{CI}_{95\%}$ of the full parameter set. See legend of Figure S7 for further details.

9 Text S9: Comparison between the HR threshold and the reinfection threshold of Gomes et al. [1]

Here we provide a contrasting discussion of our HR threshold with the reinfection threshold introduced by Gomes et al. [1, 15]. Although both thresholds indicate important qualitative changes in the epidemic dynamics, such as a sharp increase of the number of reinfections, there are several notable differences:

1. The reinfection dynamics does not operate on the same time scale and relies on different biological mechanisms. In the model of Gomes et al. [1, 15] reinfection is due to antigenic drift and allow to explain the long-term ($\sim$years)

recurrent dynamics of influenza. By contrast, we are interested in short-term ($\sim$weeks or months) HRs caused by the same strain due to immune failure.

2. Structurally these models are different. The model of Gomes et al. [1, 15] assumes that all infected individuals develop a long term immunity following infection but this immunity confers only partial protection against reinfection. By contrast, our model assumes that a fraction α of the infected individuals pass through a window of susceptibility before they develop a fully protective immunity against reinfection while the remaining proportion $(1-\alpha)$ returns to the susceptible class. As such, one can not deduce one model from the other.

3. Without demographic processes (birth-death) the equilibrium dynamics differ. Below their respective thresholds both models have a unique, disease free, equilibrium. Above the reinfection threshold, the model of Gomes et al. tends to an endemic equilibrium whereas our model is doomed to extinction once $\alpha > 0$. This is because each time a recovered host passes through the W stage, he has a non-zero probability to enter and remain in the H stage.

In summary, the reinfection threshold in the model of Gomes et al. is a threshold of long-term, sustained reinfection whereas our threshold corresponds to a threshold of short-term, limited HR.

10 Text S10: Partial susceptibility in the W stage

Partial susceptibility in the W stage can be modelled by multiplying the susceptibility by a factor $\sigma_W \in [0,1]$, where $\sigma_W = 1$ corresponds to full susceptibility. Here we show that the data do not allow us to estimate this additional parameter. Indeed, we included partial susceptibility in the SEICWH model and performed a log-likelihood profile on σ_W (Figure S9, upper panel) which reveals an identifiability issue as $\text{CI}_{95\%} = [0-1]$. As shown in Figure S9 (middle and lower panels), this is due to strong correlations between the partial susceptibility (σ_W), the expected duration of the window of susceptibility (ω^{-1}) and the probability to develop a protective humoral response (α). More precisely, as partial susceptibility decreases this is compensated by an increase of ω^{-1}, so that individuals stay longer in the window of susceptibility to initiate the reinfection wave. However, as the partial susceptibility approaches 0, the window of susceptibility has no more effect on the reinfection dynamics so that α decreases rapidly in order to generate the reinfection dynamics.

We tried to solve this identifiability issue by fixing $\alpha \simeq 0.8$ since this is the only parameter with available empirical estimates. Unfortunately, the log-likelihood profile of Figure S10 reveals that the $\text{CI}_{95\%}$ of σ_W remains wide and still includes 1 ($\text{CI}_{95\%} = [0.12-1]$), which suggests a more parsimonious model without partial susceptibility.

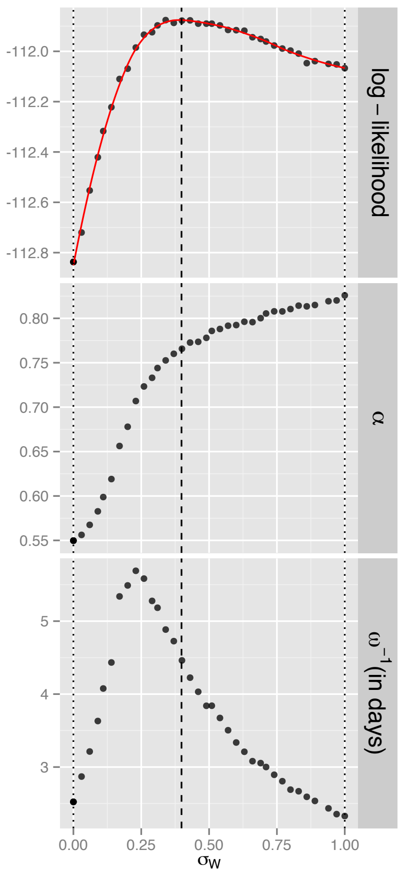

Figure S9: Analysis of the effect of assuming partial protection ($= 1 - \sigma_W$) while in the window of susceptibility. Upper panel: log-likelihood profile of σ_W. We obtain $\sigma_{W,MLE} = 0.38$ but note the identifiability issue as $\text{CI}_{95\%} : [0 - 1]$ (dotted lines). Middle panel: correlation between σ_W and the probability α to develop long-term humoral protection. Lower panel: correlation between σ_W and the expected duration ω^{-1} of the window of susceptibility.

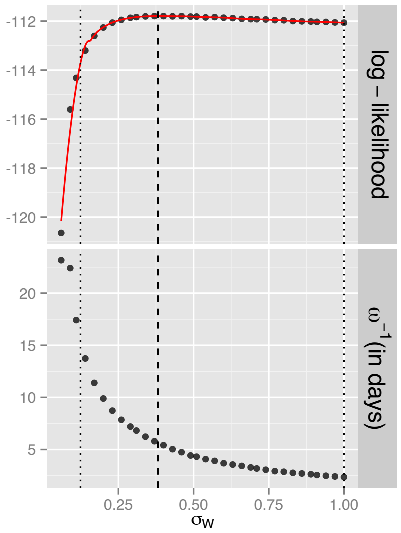

Figure S10: Analysis of the effect of fixing $\alpha = 0.83$ when estimating σ_W. The log-likelihood profile of σ_W (upper panel) shows that $\text{CI}_{95\%} : [0.12 - 1]$ remains wide, which is due to a strong correlation between σ_W and ω^{-1} (lower panel).

11 Text S11: Historical data

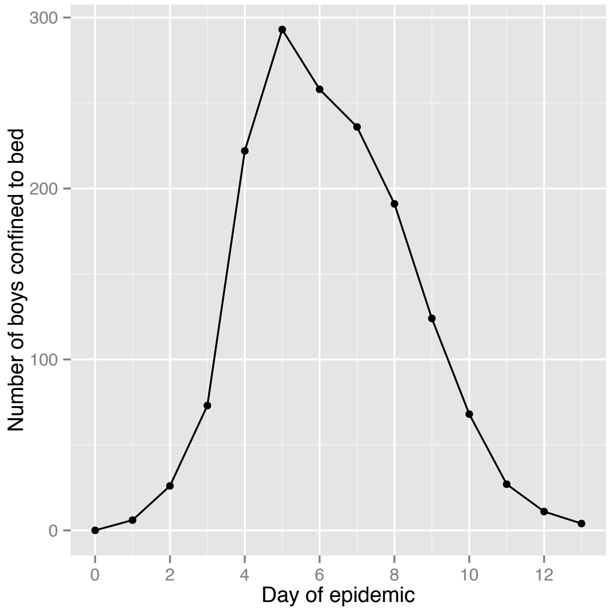

Figure S11: Data set of an influenza outbreak in a boarding school of the north of England that occurred in 1978 (reproduced from [16]). There was 763 boys between the ages of 10 and 18, all except 30 being full boarders. The staff (130 adults) were from the surrounding villages.

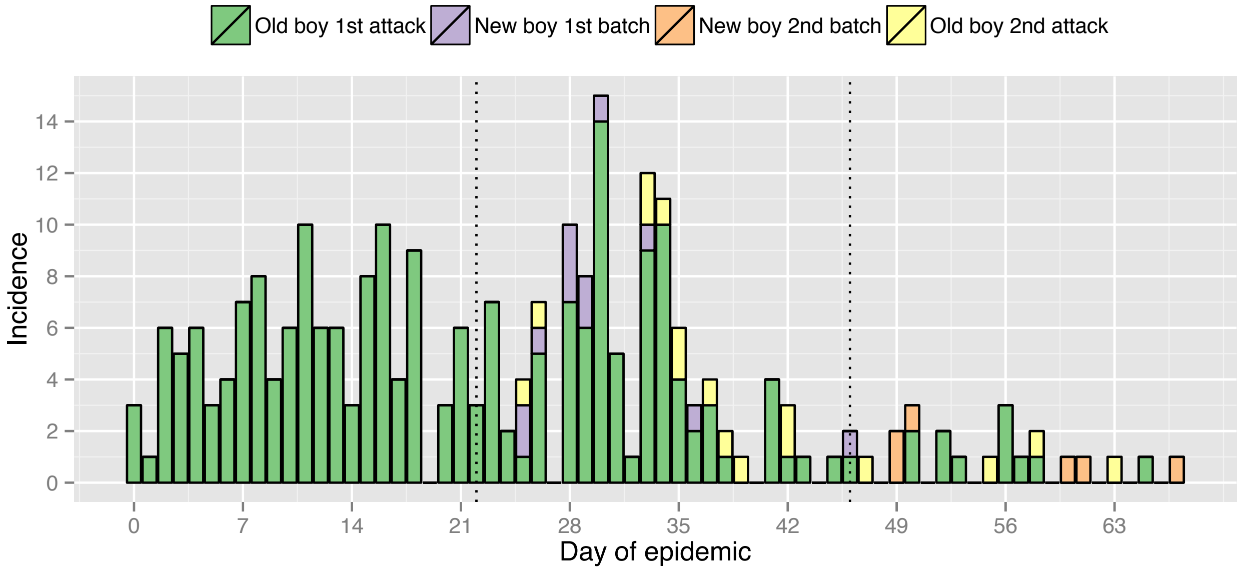

Figure S12: Diagram of 1924 influenza epidemic at the Royal Navy School, Greenwich, showing the effect of introducing fresh susceptible material in increasing incidence and second attacks among the old material (reproduced from [17]).

References

[1] Gomes MGM, White LJ, Medley GF (2004) Infection, reinfection, and vaccination under suboptimal immune protection: epidemiological perspectives. *J Theor Biol* 228:539–549.

[2] Holmgren J, Czerkinsky C (2005) Mucosal immunity and vaccines. *Nature Medicine* 11:S45–S53.

[3] Couch RB, Kasel JA (1983) Immunity to influenza in man. *Annual Reviews in Microbiology* 37:529–549.

[4] Tamura Si, Kurata T (2004) Defense mechanisms against influenza virus infection in the respiratory tract mucosa. *Jpn. J. Infect. Dis.* 57:236–247.

[5] Cox RJ, Brokstad KA, Ogra P (2004) Influenza virus: immunity and vaccination strategies. Comparison of the immune response to inactivated and live, attenuated influenza vaccines. *Scand. J. Immunol.* 59:1–15.

[6] Miller E, et al. (2010) Incidence of 2009 pandemic influenza A H1N1 infection in England: a cross-sectional serological study. *Lancet* 375:1100–1108.

[7] Murphy BR, Clements ML (1989) The systemic and mucosal immune response of humans to influenza A virus. *Curr Top Microbiol.*

[8] Subbarao K, Murphy BR, Fauci AS (2006) Development of Effective Vaccines against Pandemic Influenza. *Immunity* 24:5–9.

[9] Fraser C, Cummings DAT, Klinkenberg D, Burke DS, Ferguson NM (2011) Influenza Transmission in Households During the 1918 Pandemic. *Am J Epidemiol* 174:505–514.

[10] Smith DJ (2004) Mapping the Antigenic and Genetic Evolution of Influenza Virus. *Science* 305:371–376.

[11] Koelle K, COBEY S, Grenfell BT, Pascual M (2006) Epochal Evolution Shapes the Phylodynamics of Interpandemic Influenza A (H3N2) in Humans. *Science* 314:1898–1903.

[12] Potter CW, Jennings R, Nicholson K, Tyrrell DA, Dickinson KG (1977) Immunity to attenuated influenza virus WRL 105 infection induced by heterologous, inactivated influenza A virus vaccines. *J Hyg* 79:321–332.

[13] Park AW, et al. (2009) Quantifying the Impact of Immune Escape on Transmission Dynamics of Influenza. *Science* 326:726–728.

[14] Camacho A, et al. (2011) Explaining rapid reinfections in multiple-wave influenza outbreaks: Tristan da Cunha 1971 epidemic as a case study. *Proc. R. Soc. B* 278:3635–3643.

[15] Gomes MGM, White LJ, Medley GF (2005) The reinfection threshold. *J Theor Biol* 236:111–113.

[16] Anonymous (1978) Influenza in a boarding school. *Br Med J* 1:587.

[17] Dudley S (1926) The spread of "Droplet Infection" in Semi-isolateed Communities., (Oxford), Technical Report 111.
